# Insecticide-treated bed net utilization and associated factors among pregnant women in Ethiopia: a systematic review and meta-analysis

**DOI:** 10.1186/s12936-023-04655-7

**Published:** 2023-08-02

**Authors:** Gizachew Ambaw Kassie, Getachew Asmare Adella, Amanuel Yosef Gebrekidan, Natnael Atnafu Gebeyehu, Molalegn Mesele Gesese, Endeshaw Chekol Abebe, Misganaw Asmamaw Mengstie, Mohammed Abdu Seid, Kirubel Dagnaw Tegegne, Sefineh Fenta Feleke, Tadesse Asmamaw Dejenie, Berihun Bantie, Natnael Moges, Yenealem Solomon Kebede, Melkamu Aderajew Zemene, Anteneh Mengist Dessie, Denekew Tenaw Anley, Yordanos Sisay Asgedom

**Affiliations:** 1 School of Public Health, College of Health Science and Medicine, Wolaita Sodo University, Wolaita Sodo, Ethiopia; 2 School of Midwifery, College of Health Science and Medicine, Wolaita Sodo University, Wolaita Sodo, Ethiopia; 3 Department of Biochemistry, College of Health Sciences, Debre Tabor University, Debre Tabor, Ethiopia; 4 Unit of Physiology, Department of Biomedical Science, College of Health Science, Debre Tabor University, Debre Tabor, Ethiopia; 5 Department of Nursing, College of Medicine and Health Science, Wollo University, Dessie, Ethiopia; 6 Department of Public Health, College of Health Sciences, Woldia University, Woldia, Ethiopia; 7 Department of Medical Biochemistry, College of Medicine and Health Sciences, University of Gondar, Gondar, Ethiopia; 8 Department of Comprehensive Nursing, College of Health Sciences, Debre Tabor University, Debre Tabor, Ethiopia; 9 Department of Pediatrics and Child Health Nursing, College of Health Sciences, Debre Tabor University, Debre Tabor, Ethiopia; 10 Department of Medical Laboratory Science, College of Health Sciences, Debre Tabor University, Debre Tabor, Ethiopia; 11 Department of Public Health, College of Health Sciences, Debre Tabor University, Debre Tabor, Ethiopia

**Keywords:** Insecticide-treated net, Utilization, Malaria, Pregnant women, Ethiopia

## Abstract

**Background:**

Malaria infection during pregnancy endangers the pregnant woman, fetus, and newborn child. Thus, the use of an insecticide-treated net (ITN) is one of the most frequently advised methods for preventing malaria during pregnancy. Contrary findings have been reported in various studies on ITN utilization among pregnant women in Ethiopia. Therefore, this study was aimed to estimate the pooled prevalence of ITN utilization and its associated factors among pregnant women in Ethiopia.

**Methods:**

Published articles from PubMed, Google Scholar, Science Direct, AJOL and Cochrane library were systematically searched. All cross-sectional studies on ITN utilization among pregnant women were included in this meta-analysis. To estimate the pooled prevalence and odds ratio, a random-effect model was used; and a subgroup analysis was performed to identify the possible source of heterogeneity. Begg’s and Egger’s tests were used to identify possible publication bias.

**Results:**

Ten cross-sectional studies with 7,161 participants were included. The pooled prevalence of ITN utilization among all pregnant women who had access to ITN in Ethiopia was 59.42% (95% CI 51.14, 67.69). Statistically significant heterogeneity was observed (I^2^ = 97.7%; p < 0.0001). Higher educational status (OR = 3.47, 95% CI   2.32, 5.2), pregnant women who had antenatal care visits (OR = 2.37, 95% CI 1.97, 2.65) and having good knowledge of malaria prevention practices (OR = 10.63, 95% CI   5.31, 21.29) were associated with ITN utilization among pregnant women.

**Conclusion:**

The utilization of ITNs among pregnant women was much lower than the national target. Higher education status, attending ANC and a good level of knowledge were found to be independent predictors of ITN utilization. Improving women’s understanding of ITNs will enhance their use, and the government and health sectors should encourage pregnant mothers to enroll in antenatal care.

**Supplementary Information:**

The online version contains supplementary material available at 10.1186/s12936-023-04655-7.

## Background

Malaria is a preventable and treatable disease that continues to have a devastating impact on people’s health and livelihoods all over the world. By 2021, nearly half of the world’s population was at risk of malaria, with 247 million new cases and 619,000 malaria-related deaths [1]. Some populations are at significantly higher risk of contracting malaria and developing severe disease, including infants, children under the age of five, and pregnant women [1, 2]. In 2021, there were an estimated 40 million pregnancies in 38 African countries with moderate to high transmission, of which 13.3 million (33%) were exposed to malaria during pregnancy. The highest prevalence of malaria exposure during pregnancy was (40.7%) in West Africa, followed by (39.8%) in Central Africa, and 20% in East and Southern Africa [1].

Malaria infection during pregnancy endangers the pregnant woman, fetus, and newborn child. It is frequently associated with anaemia, Intrauterine Growth Restriction (IUGR), and complications such as low birth weight and trans placental parasitaemia [3]. For instance, malaria causes one-fourth of all severe maternal anaemia cases and 20% of all low-birthweight babies in malaria-endemic areas [2]. In sub-Saharan Africa (SSA), placental malaria infection is estimated to cause 900,000 low birth weight deliveries each year owing to intrauterine growth retardation and preterm delivery [4]. An estimated 200,000 infant deaths and 100,000 neonatal deaths occur annually, with 18% of global neonatal mortality attributable to malaria [5].

The use of insecticide-treated bed nets (ITN), intermittent preventive therapy with sulfadoxine-pyrimethamine (IPTp-SP), and early case detection are methods for preventing malaria during pregnancy [6]. Therefore, it is recommended that all pregnant women sleep under an ITN as early as possible during pregnancy, although ideally before becoming pregnant. Providing an ITN at the first contact will help to keep the pregnant woman and her fetus safe from malaria [7].

Globally, the percentage of pregnant women sleeping under an ITN has increased considerably between 2000 and 2021, from 3 to 53%. However, overall access to and use of ITNs have continued to decline in SSA including Ethiopia, since 2017 [1]. According to a malaria epidemiological and interventional study conducted in Ethiopia, national household ITN coverage was 64% [9]. Similarly, in Ethiopia, the utilization of ITN among pregnant women ranges from 33.6 to 78.8% [10, 11]. The most common factors frequently associated with ITN utilization reported in Ethiopian studies were educational level, place of residence, attending ANC, distance from health care facility, media exposure of the mothers, awareness levels, and ITN-related factors, such as ITN accessibility, sufficiency, quality, physical condition, maintenance, replacement, and effectiveness [10–15].

The utilization of ITN in Ethiopia has been the subject of various epidemiological studies, but the findings have been inconsistent and vary significantly. These separate studies indicated that the prevalence of ITN utilization among pregnant women in Ethiopia ranges from 33.6 to 78.8% [10–15]. Such a disparity in the magnitude and determinants of ITN utilization may not be satisfactory for policymakers and planners to address the issue of low ITN utilization with such wide disparities. As a result, policymakers need to understand the heterogeneity in ITN utilization across regions and districts, as well as the factors that affect this heterogeneity in ITN utilization. Therefore, this systematic review and meta-analysis aimed to calculate the pooled estimate of ITN utilization among pregnant women in Ethiopia.

This study provides real evidence that policymakers and programme directors may use to develop effective interventions to increase the utilization of ITNs by pregnant women and lower the rate of malaria infection during pregnancy.

## Methods

### Searching strategy and study identification

A systematic review and meta-analysis was conducted to estimate the magnitude of insecticide-treated bed net utilization and associated factors among pregnant women in Ethiopia. All published research reports on insecticide-treated bed net utilization and associated factors among pregnant women in Ethiopia were retrieved from electronic databases, including Medline, Google Scholar, and Science Direct, AJOL, and Cochrane Library. All databases were searched for articles published before October 30, 2022. The searching strategy for published articles was country-specific (studies conducted only in Ethiopia). The key words used in the search were “utilization,“ “of insecticide-treated bed net” “long lasting integrated bed net” “among pregnant women in Ethiopia,“ “associated factors” and “Ethiopia.“ The search terms were used individually as well as in combination using “OR” or “AND” (Additional file 1). This systematic review and meta-analysis included all articles published up to October 30, 2022. This systematic review and meta-analysis was reported based on the guideline of the Preferred Reporting Items for Systematic Reviews and Meta-Analyses (PRISMA) [16] (Additional file 2).

### Inclusion criteria

#### Population

A study was carried out on pregnant women who owned ITN.

#### Study setting

Studies conducted across all regions of Ethiopia were considered.

#### Publication

Both published and unpublished articles were considered for this review and meta-analysis. Unpublished studies were retrieved from the official website of Addis Ababa University electronic database.

#### Study design and language

All original studies published in English in Ethiopia that reported the extent of insecticide-treated bed net utilization among pregnant women in Ethiopia were included.

### Exclusion criteria

This study excluded the studies with difficult access to full text and studies that did not report the primary outcomes of interest.

### Outcome measurement

This systematic review and meta-analysis have two main objectives. The first objective was to estimate the pooled prevalence of ITN utilization among pregnant women in Ethiopia. The second objective was to identify factors associated with ITN utilization among pregnant women in Ethiopia. ITN utilization was measured as the proportion of a given population group that slept with an ITN the night before the survey who had at list one ITN in their household. The second outcome of this study was the most frequently associated factors with ITN utilization. The input variables required by “metan” contained the cells of the 2 × 2 tables, that is, the number of pregnant women who did and did not utilize ITN in the exposed and non-exposed groups in each study. Maternal age, educational status, place of residence, started ANC visits, monthly income, malaria prevention knowledge and distance from health facilities were potential variables which selected in the analysis.

### Data abstraction

All studies retrieved from all databases were imported into Endnote version X7, and duplicate articles were manually removed. Two independent reviewers (GAK and AYG) screened all articles for eligibility criteria: initially, the titles and abstracts of the articles were screened, followed by the full text. If agreement could not be reached regarding study selection, disagreements were settled by inviting a third investigator (GAA). The data were then extracted from the included articles using Excel spreadsheet software. The data extraction format included the authors name, year of publication, study setting, region where the study was conducted, sample size, total number of cases, proportion of insecticide-treated bed net utilization,, study period, study area, study population, quality rating and factors (maternal age, level of education, place of residence, started ANC visits, monthly income, level of knowledge on malaria prevention practice, and distance from health facilities with their cross-tabulation to ITN utilization (a, b, c, d)).

### Quality assessments

Joanna Briggs Institute (JBI) tool adapted for cross-sectional studies was used to assess quality of each study [17]. The following criteria for evaluation are included in the tool: appropriateness of the source population list, proper recruitment of study participants, sample size sufficiency, and appropriateness of the study area and subject description, data analysis with sufficient coverage of the sampled data analysis, measurement of the condition using a standard, reliable, and consistent approach for all participants, suitability of statistical analysis, adequate response rate, or use of appropriate handling mechanism for low response rate. Each study was critically evaluated by two independent reviewers. Disagreements among reviewers were settled through discussion. If they did not agree, a third reviewer was involved in resolving the disagreements between independent reviewers. Subsequently, studies were included in the final systematic review and meta-analysis if they received a total score of ≥ 50% on the quality evaluation checklist criteria (Table 1) [18].

### Statistical analysis

After extraction, the data were entered into a computer using Excel spreadsheet software and exported to STATA 14 (Stata Corp, USA) software for analysis [37]. A forest plot was used to check for heterogeneity among the included studies using I-squared (I^2^) test statistics. I-squared statistical heterogeneity test is considered statistically significant at p ≤ 0.05. I^2^ values of 0%, 25%, 50%, and 75% were assumed to represent no, low, medium, and high heterogeneity, respectively [19]. The pooled utilization of ITN among pregnant women was estimated using the Freeman–Tukey double arcsine transformation method with the DerSimonian and Laird random-effects model because of the high degree of heterogeneity observed (I^2^ > 97.7%, P < 0.0001). To indicate the presence of heterogeneity, a forest plot was generated. To examine potential differences across studies and identify sources of variation, subgroup analysis and meta-regression were performed based on region and study setting. Both Egger’s and Begg’s tests were used to look for evidence of publication bias, with a p-value of less than 0.05 used as a cut-off point. A leave-one-out sensitivity analysis was also performed to evaluate the main studies that had the greatest impact on the between study heterogeneity. By excluding each study one at a time, an analysis was performed to assess the effect of each study on the pooled estimate of ITN utilization. For the second outcome, all most frequently associated factors with ITN utilization in the multivariate analysis results of primary studies were included in the analysis. A random effect meta-analysis model was used to estimate the pooled odds ratio of the association between each factor with ITN utilization. Finally, the study findings were presented by using forest plots with corresponding prevalence and 95% confidence intervals.

## Results

### Selection of studies

**Fig. 1:**
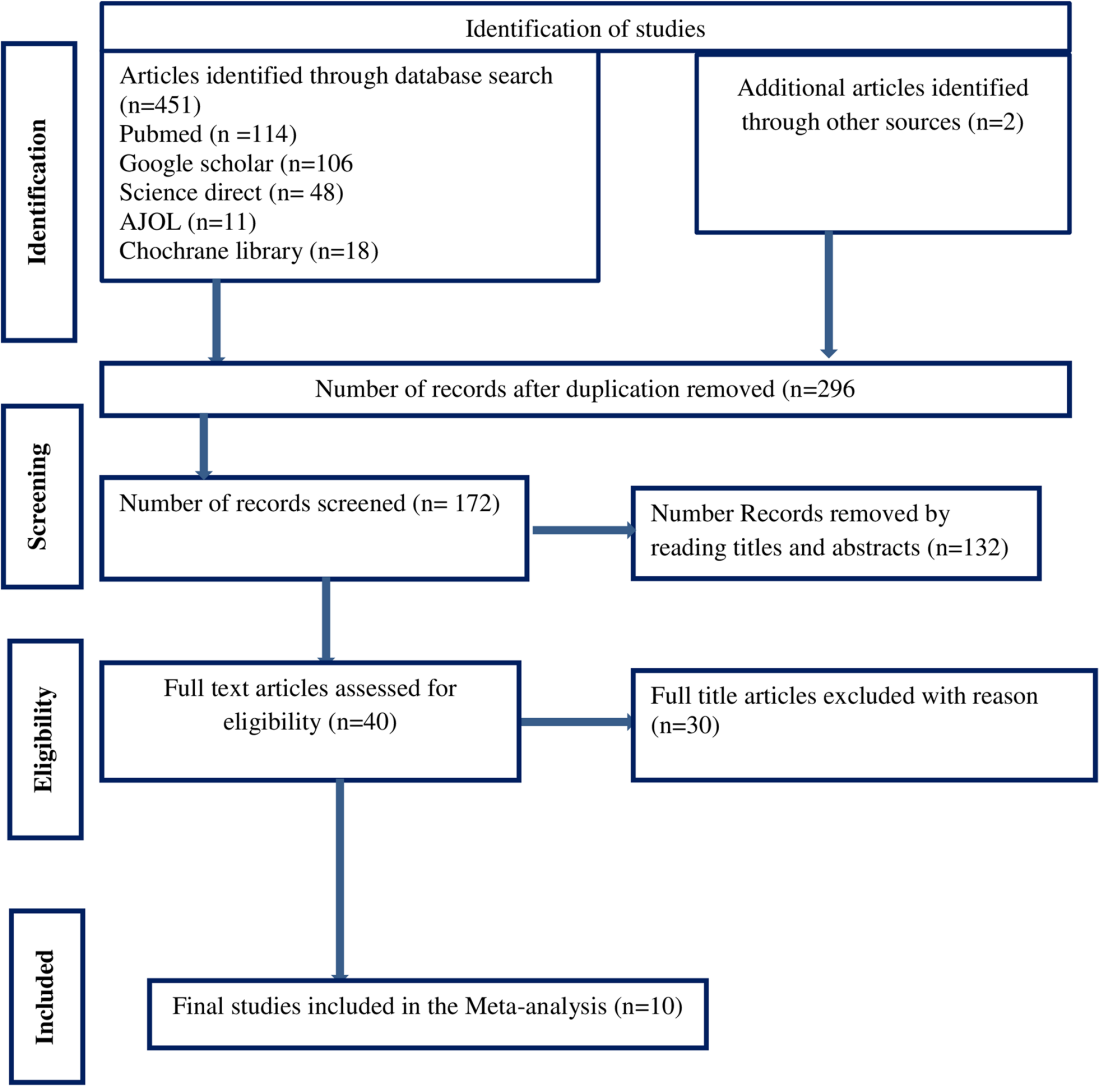
PRISMA floe diagram of articles screened and the selection process on insecticide treated net utilization and associated factors among pregnant women in Ethiopia, 2022

A total of 453 published and unpublished studies were identified through database search. A total of 281 studies were duplicates and were removed, while 172 were advanced to the screening stage. Then, 132 studies were excluded based on title and abstract screening, leaving 40 full articles. Finally, ten studies met the eligibility requirements and were included in the final analysis to estimate the overall prevalence of ITN use among pregnant women. The detailed selection procedure is illustrated in (Fig. 1).

### Characteristics of identified studies

This systematic review and meta-analysis included 10 cross-sectional studies in total [10–15, 20–23]. This meta-analysis included 7,161 pregnant women who owned at least one ITN in their households. The years of publication ranged from 2010 to 2022. The minimum and maximum sample sizes were 151 and 3,784, respectively, from studies conducted in the South Nations, Nationalities, and Peoples’ Region (SNNPR) and Oromia, respectively [22, 23]. Concerning the distribution of studies across the country’s regions, three were from the Amhara region [11, 14, 15], two from the Oromia region [13, 22], two from Tigray [20, 21], and three from SNNPR [10, 12, 23] (Table 2).

**Table 1:** Quality assessment for the included Studies using JBI standardized checklist

Items	Appropriateness of the source population list	Describe study setting and participant	Valid and reliable exposure measurement	Objective and standard criteria for measurement	Identified confounder	Strategies to deal with confounders	Valid and reliable outcome measurement	Appropriate statically analysis	No of ‘yes’ ‘
Shonga et al. [12]	Yes	Yes	No	Yes	Yes	Yes	Yes	Yes	7/8 = 87.5
Yirsaw et al. [14]	Yes	Yes	Yes	Yes	No	No	Yes	Yes	6/8 = 75
Yitayew et al. [15]	Yes	Yes	No	Yes	Yes	No	Yes	Yes	6/8 = 75
Ouedraogo et al. [22]	Yes	Yes	No	Yes	Yes	Yes	Yes	Yes	7/8 = 87.5
Angesom et al. [20]	Yes	Yes	No	Yes	Yes	Yes	Yes	Yes	7/8 = 87.5
Belay et al. [21]	Yes	Yes	Yes	Yes	No	No	Yes	Yes	6/8 = 75
Tariku et al. [11]	Yes	Yes	Yes	Yes	No	No	Yes	Yes	6/8 = 75
Tesfaye et al. [13]	Yes	Yes	No	Yes	Yes	Yes	Yes	No	6/8 = 75
Yeshaneh et al. [23]	Yes	Yes	Yes	Yes	No	Yes	Yes	No	6/8 = 75
Nadew et al. [10]	Yes	Yes	No	Yes	Yes	Yes	Yes	Yes	7/8 = 87.5

**Table 2:** Descriptive summary of 10 studies included in the meta-analysis of ITN utilization among pregnant women in Ethiopia, 2022

No	Authors	Publication Year	Study setting	Region	Study period (season )	Place Residence	Study design	Sample size	ITN utilization	Risk of bias
1	Shonga et al. [12]	2018	Damot Pulasa	SNNPR	January	Rural	CS	630	72.5	Low
2	Yirsaw et al. [14]	2021	East Belessa	Amhara	February	Rural	CS	144	54	Moderate
3	Yitayew et al. [15]	2018	Addis Zemen	Amhara	May	Urban	CS	226	70.8	Low
4	Ouedraogo et al. [22]	2019	Jimma Zone	Oromia	January	Rural	CS	3784	55	Low
5	Angesom et al. [20]	2019	Asgede Tsimbla	Tigray	January -June	Rural	CS	550	63.1	Low
6	Belay et al. [21]	2010	Raya Azebo	Tigray	May- June	Rural	CS	439	56.7	Moderate
7	Tariku et al. [11]	2020	Awabel Woreda	Amhara	June	Rural	CS	422	33.6	Moderate
8	Tesfaye et al. [13]	2022	Miesso District	Oromia	April	Rural	CS	424	39.9	Low
9	Yeshaneh et al. [23]	2020	Halaba Kulito	SNNPR	April	Urban	CS	151	70.8	Moderate
10	Nadew et al. [10]	2016	Sodo Zuria	SNNPR	April	Rural	CS	435	78.39	Low

A total of five articles reported that educational level were significant associated with ITN utilization among pregnant women [12, 15, 21–23]. Furthermore, four studies were reported that a pregnant women started ANC visit [10, 11, 13, 22]; three studies reported level of knowledge on malaria prevention practice during pregnancy [10–12]; three studies reported place of residence [13, 14, 20]; two studies reported distance from health facility [11, 23]; two studies reported monthly income [13, 15] and three studies reported maternal age [10, 11, 15] which were significant associated factors of ITN utilization among pregnant women in Ethiopia.

### Pooled prevalence of ITN utilization among pregnant in Ethiopia (meta-analysis)

**Fig. 2:**
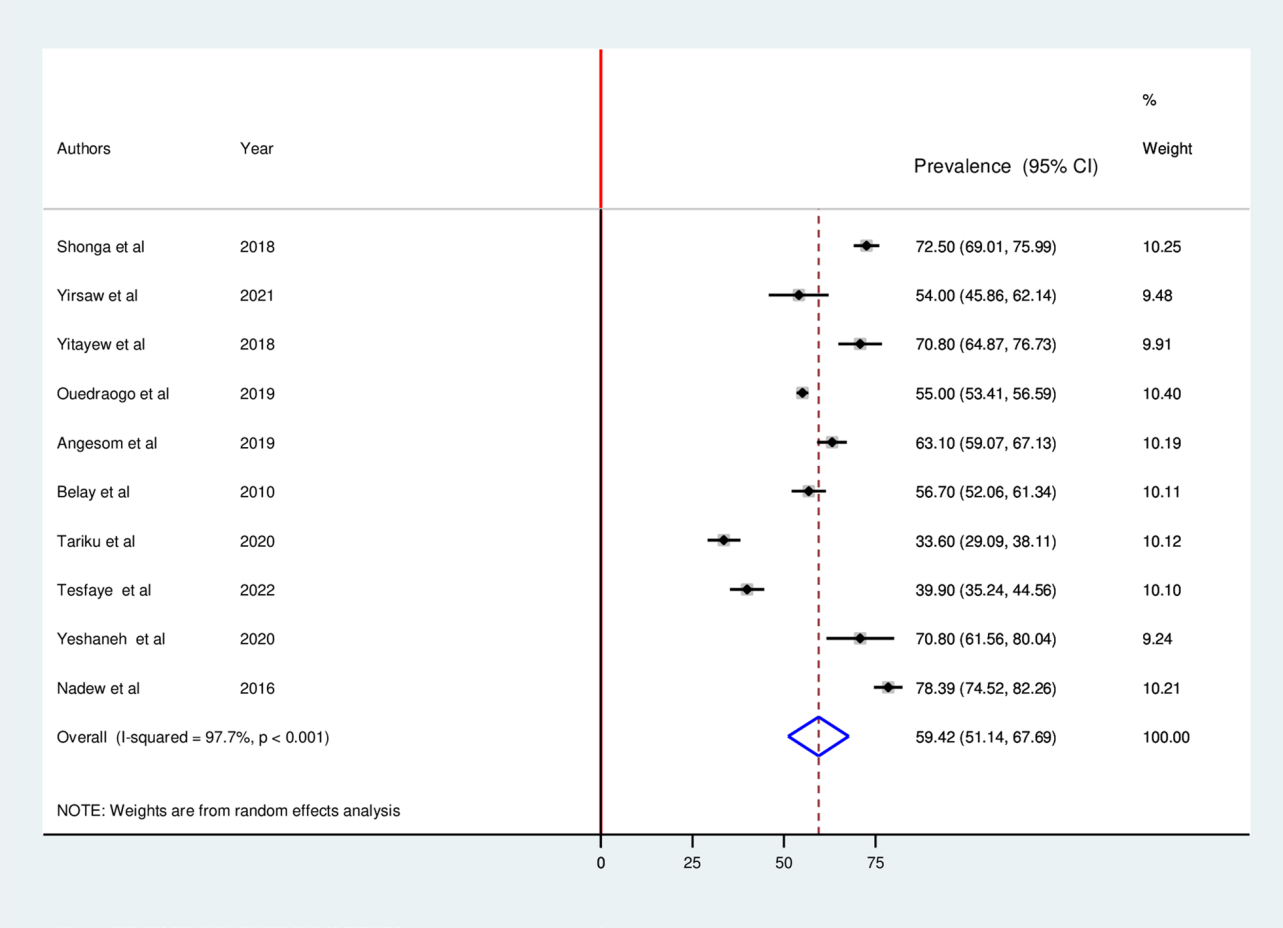
Forest plot displaying pooled prevalence ITN utilization among pregnant in Ethiopia, 2022

The pooled prevalence of ITN utilization among pregnant in Ethiopia was 59.42% (95% CI 51.14, 67.69). As shown in the forest plot below, a statistically significant heterogeneity was observed (I^2^ = 97.7%; p < 0.0001) (Fig. 2). Therefore, the pooled utilization of ITN was estimated by using random-effects models. In addition, the significant magnitude of heterogeneity also indicates the need to conduct subgroup analysis to identify the sources of heterogeneity across studies. In terms of individual prevalence, Tigray and the Amhara region had the lowest (33.6%) and highest (78.39%) levels of ITN utilization, respectively.

### Sub group analysis

**Fig. 3:**
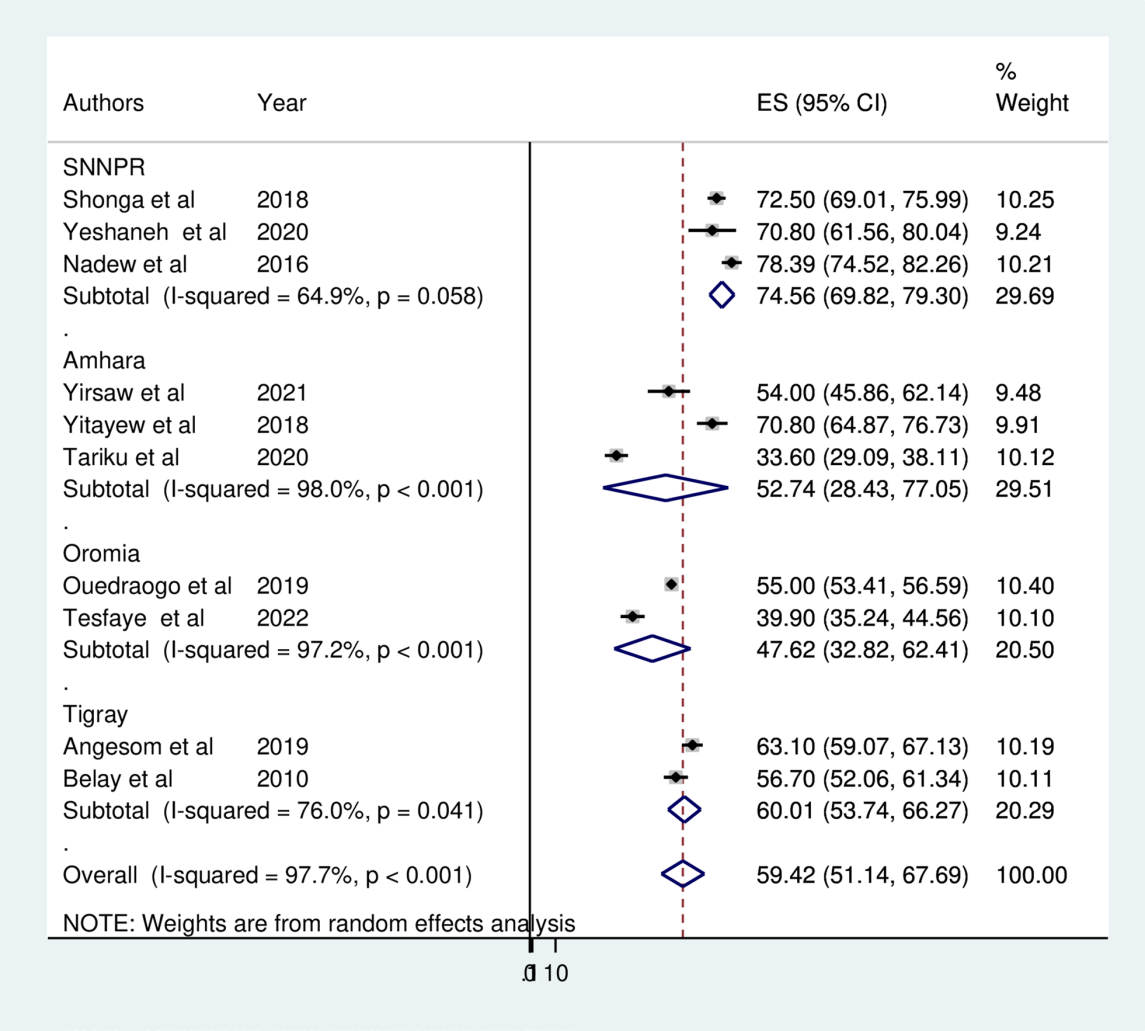
Forest plot displaying subgroup analysis by region for the pooled prevalence ITN utilization among pregnant in Ethiopia, 2022

**Fig. 4:**
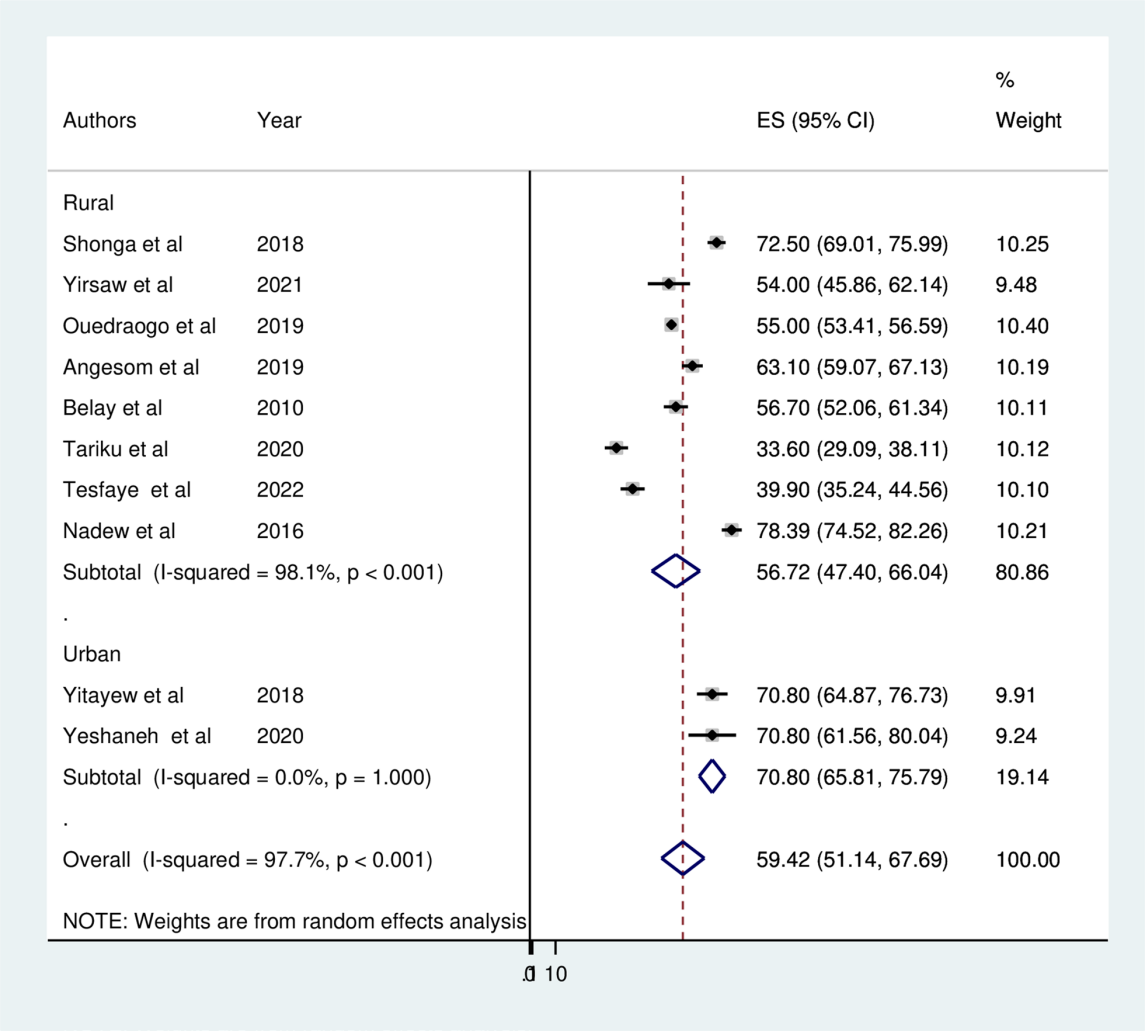
Forest plot displaying subgroup analysis by study setting for the pooled prevalence ITN utilization among pregnant in Ethiopia, 2022

**Fig. 5:**
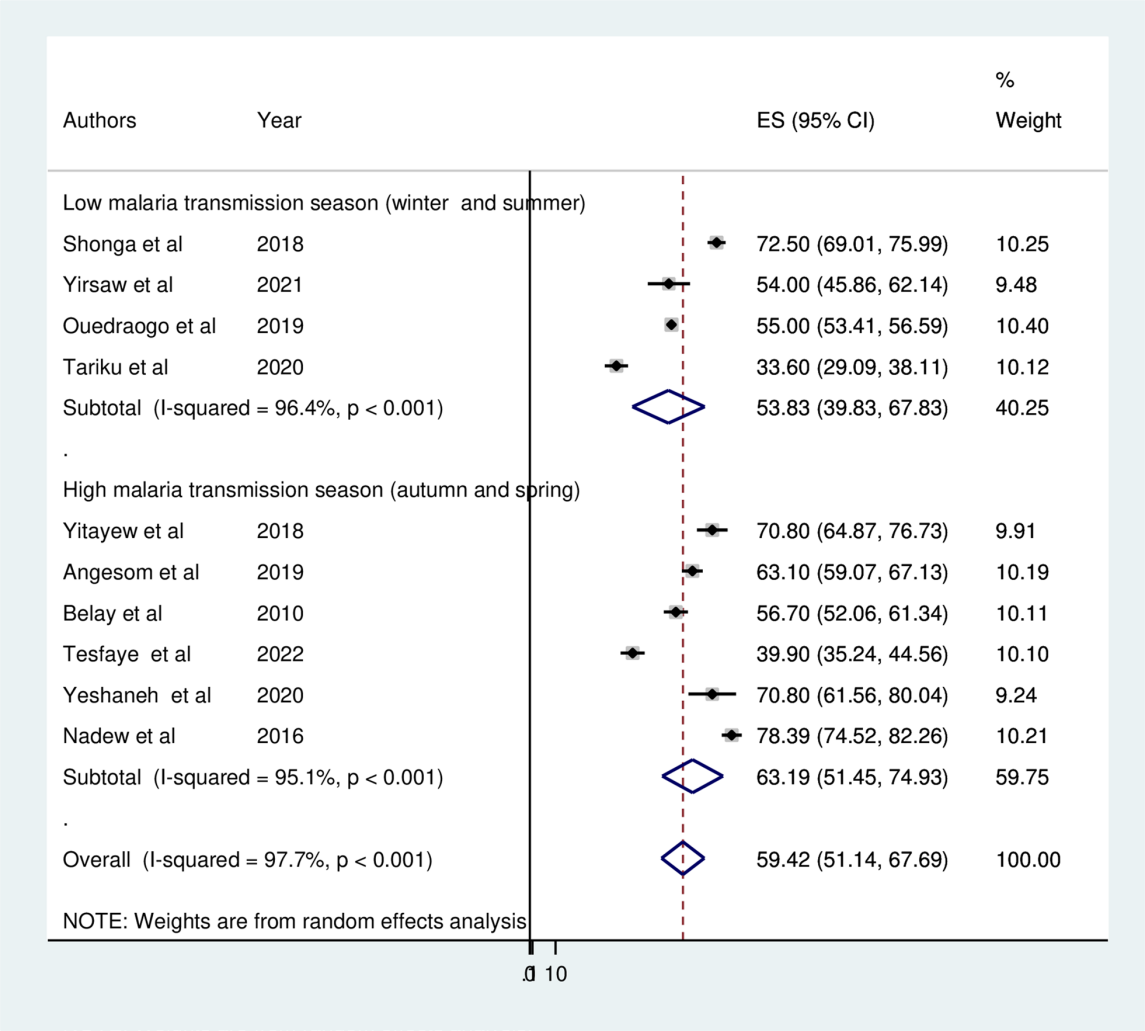
Forest plot displaying subgroup analysis by study peroid for the pooled prevalence ITN utilization among pregnant in Ethiopia, 2022

To assess the possible source of heterogeneity, subgroup analysis was performed based on the study area (regions), study period and study setting (urban/rural). As a result, statistically significant heterogeneity was observed between groups (P < 0.05). Regarding the pooled prevalence of ITN utilization among pregnant women by region where the studies were conducted the highest prevalence of ITN utilization was reported in SNNPR 74.56% (95% CI 69.82, 79.30) and the lowest was in Oromia 47.62% (95% CI 32.82, 62.41) (Fig. 3). Subgroup analysis by study setting revealed that the pooled prevalence of ITN utilization among pregnant women was 70.08% (95% CI 65.81, 75.59) in urban studies and 56.72% (95% CI  47.40, 66.04) in rural studies (Fig. 4). A subgroup analysis by study period (malaria transmission season) indicated that ITN utilization was 30% and 40% in studies conducted during the high malaria transmission season (autumn and spring) and low malaria transmission season (winter and summer), respectively (Fig. 5).

### Heterogeneity and publication bias

To determine the cause of heterogeneity, meta-regression was used with sample size and publication year as covariates. The analysis found that neither sample size nor publication year were statistically significant sources of heterogeneity (Table 3).

**Table 3:** A meta-regression analysis to determine factors affecting between-study heterogeneity of ITN utilization among pregnant women in Ethiopia

Heterogeneity source	Coefficient	Standard error	p-value
Publication year	−0.0014	0.0039	0.78
Sample size	−1.365	1.54	0.40

**Fig. 6:**
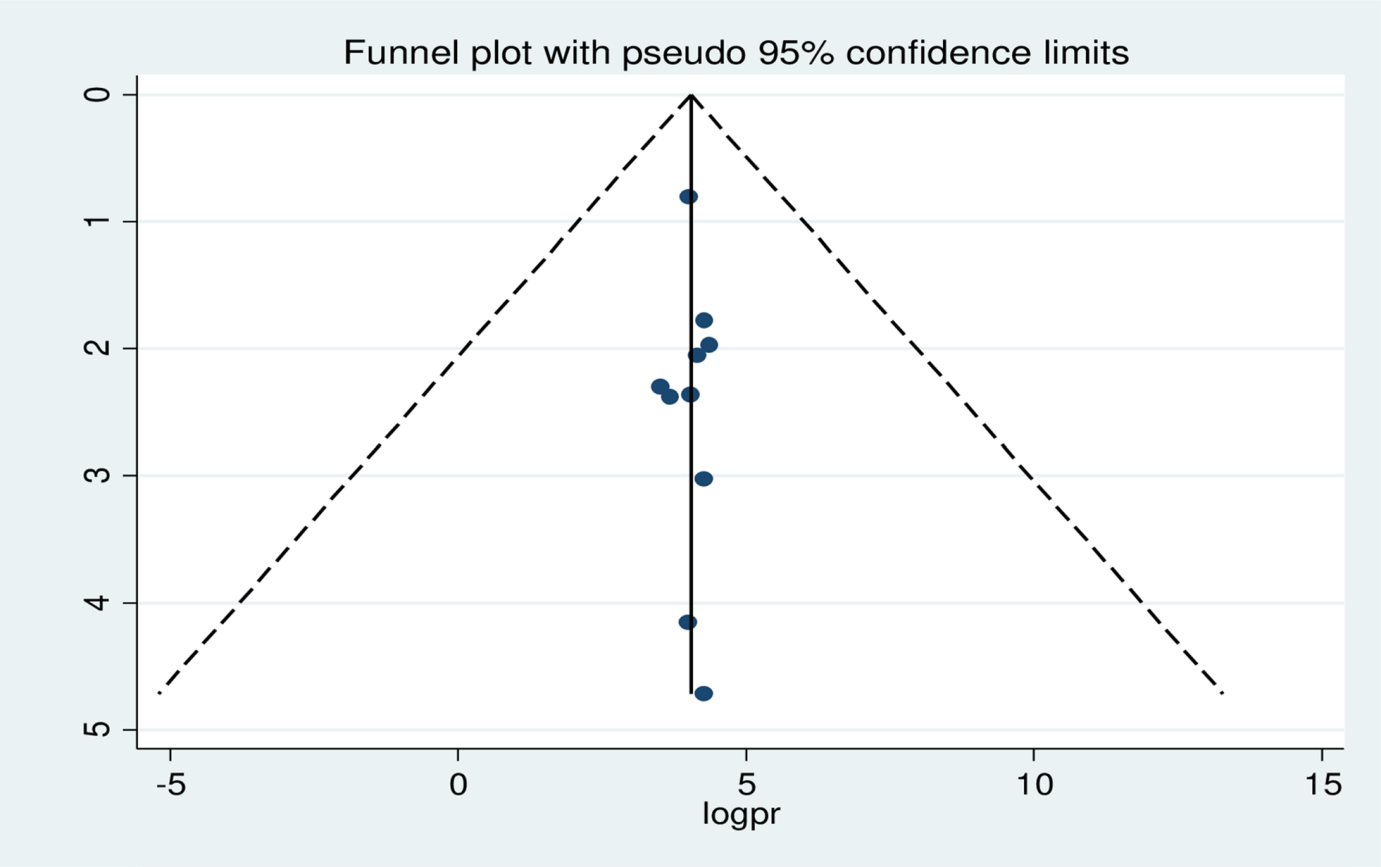
Funnel plot displaying publication bias of studies reporting the utilization ITN among pregnant women in Ethiopia, 2022

The funnel plot was visually inspected to assess potential publication bias, which was statistically supported by Begg’s and Egger’s tests. The symmetrical distribution of the included publications in a large inverted funnel indicated the absence of publication biases (Fig. 6). The Begg and Egger tests revealed no publication bias among the studies included to estimate the pooled prevalence of ITN utilization among pregnant women, with p - values of (p = 0.78) and (p = 0.87), respectively.

### Sensitivity analysis

**Fig. 7:**
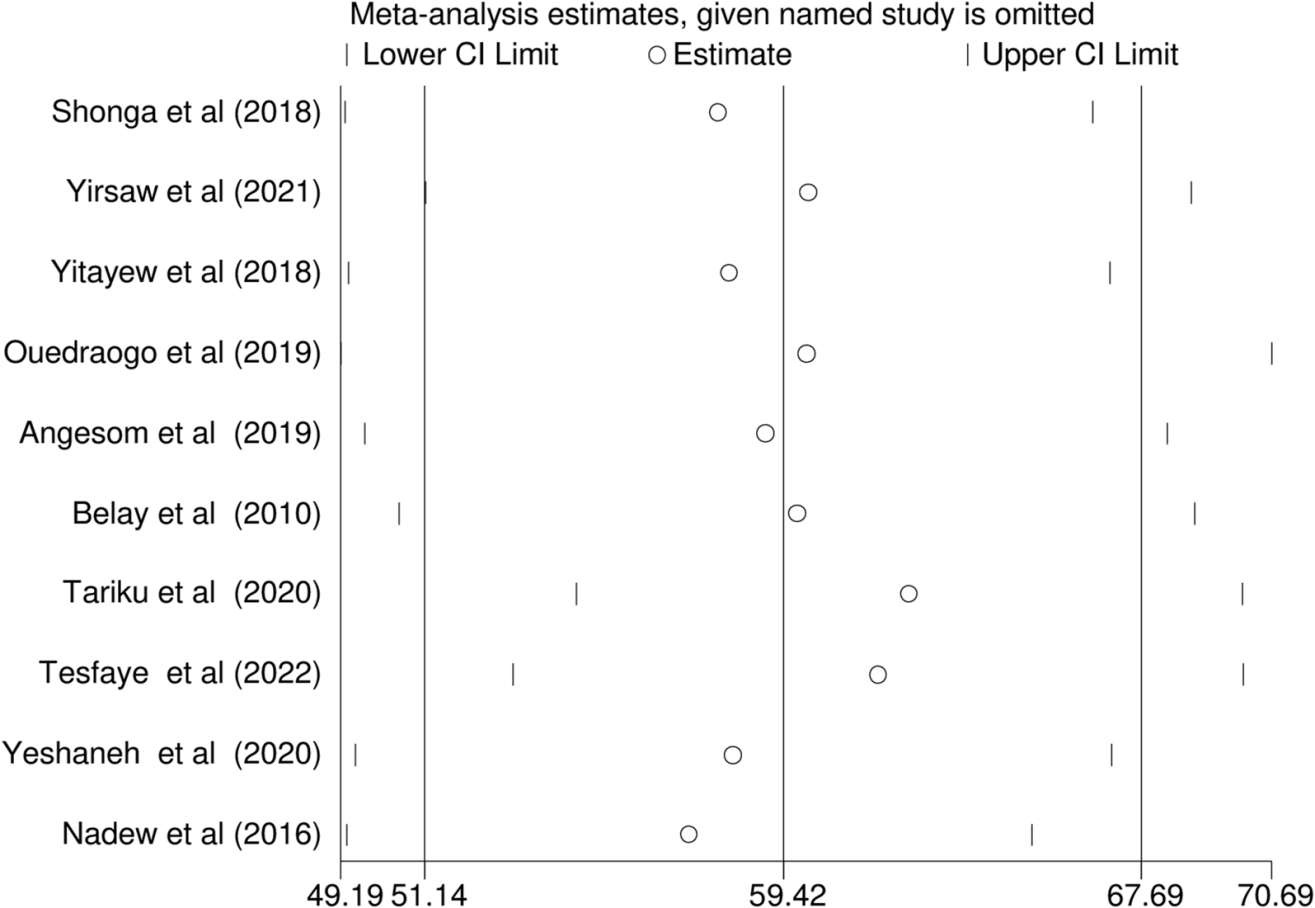
Forest plot displaying sensitivity analysis on pooled prevalence ITN utilization among pregnant in Ethiopia, 2022

Sensitivity analysis was used to assess the effect of a single study on the pooled prevalence of ITN utilization among pregnant women in Ethiopia by excluding each study one at a time. The findings revealed that no single study made a statistically significant difference in the pooled prevalence of ITN utilization (Fig. 7).

### Factors associated with ITN utilization among pregnant women

To identify factors associated with ITN utilization among pregnant women, variables such as maternal age, level of education, place of residence, started ANC visits, income, level of knowledge, and distance from health facilities were extracted from the included studies. Finally, three variables were identified as independent predictors of ITN utilization among pregnant women: educational status, attending ANC, and level of knowledge on malaria prevention practices during pregnancy.

### Association of educational status with ITN utilization

**Fig. 8:**
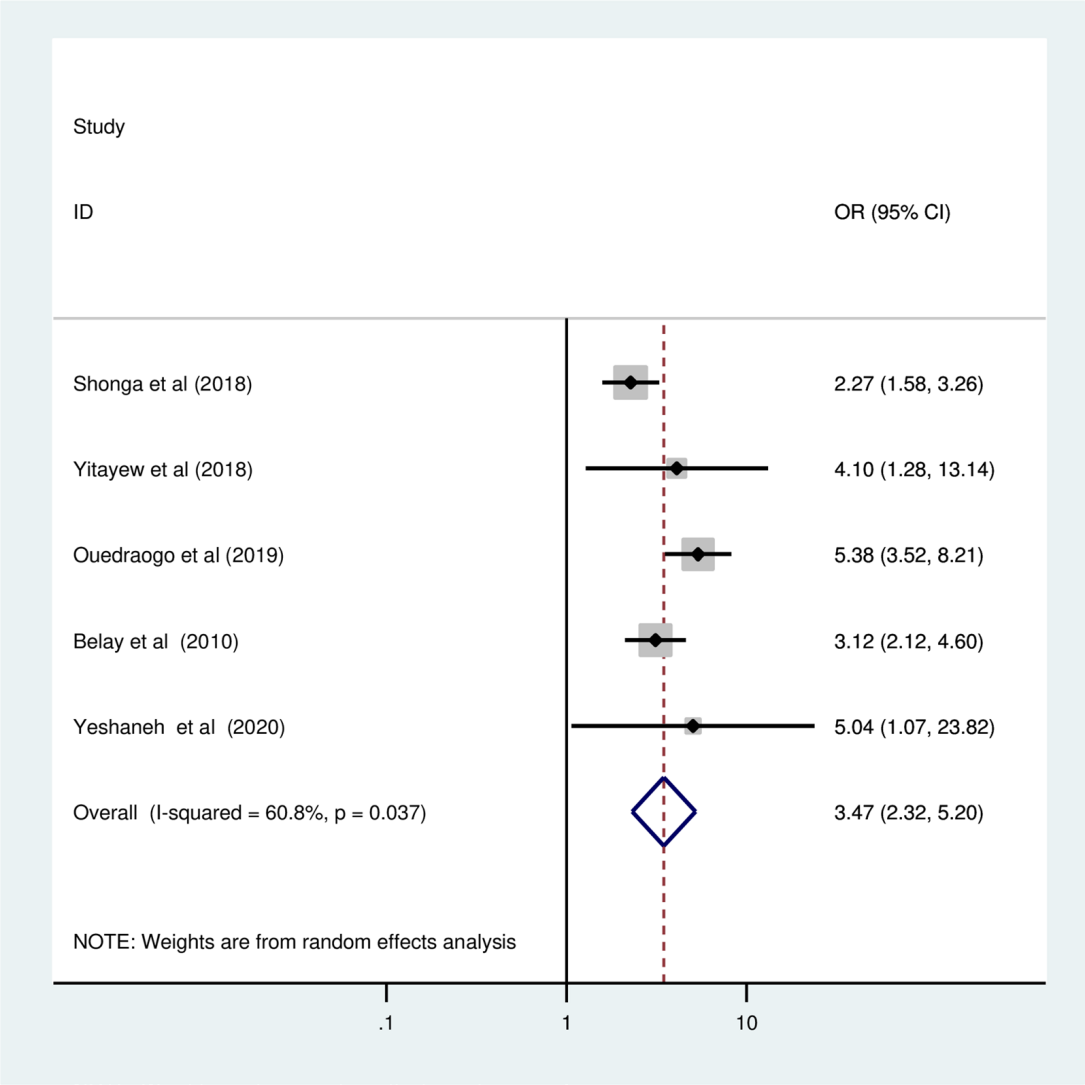
The pooled odd ratio of the association between no formal education and ITN utilization among pregnant women in Ethiopia, 2022

Five primary studies with a total 3,841 participant of reported pregnant woman who with higher educational status as a determinant of ITN utilization in pregnant women [12, 15, 21–23]. In this meta-analysis, the pooled odds ratio revealed that pregnant women with a higher educational level were 3.47 times more likely to use ITN than illiterate pregnant women (OR = 3.47, 95% CI = 2.32, 5.2) (Fig. 8).

### Association of ANC visiting and ITN utilization

**Fig. 9:**
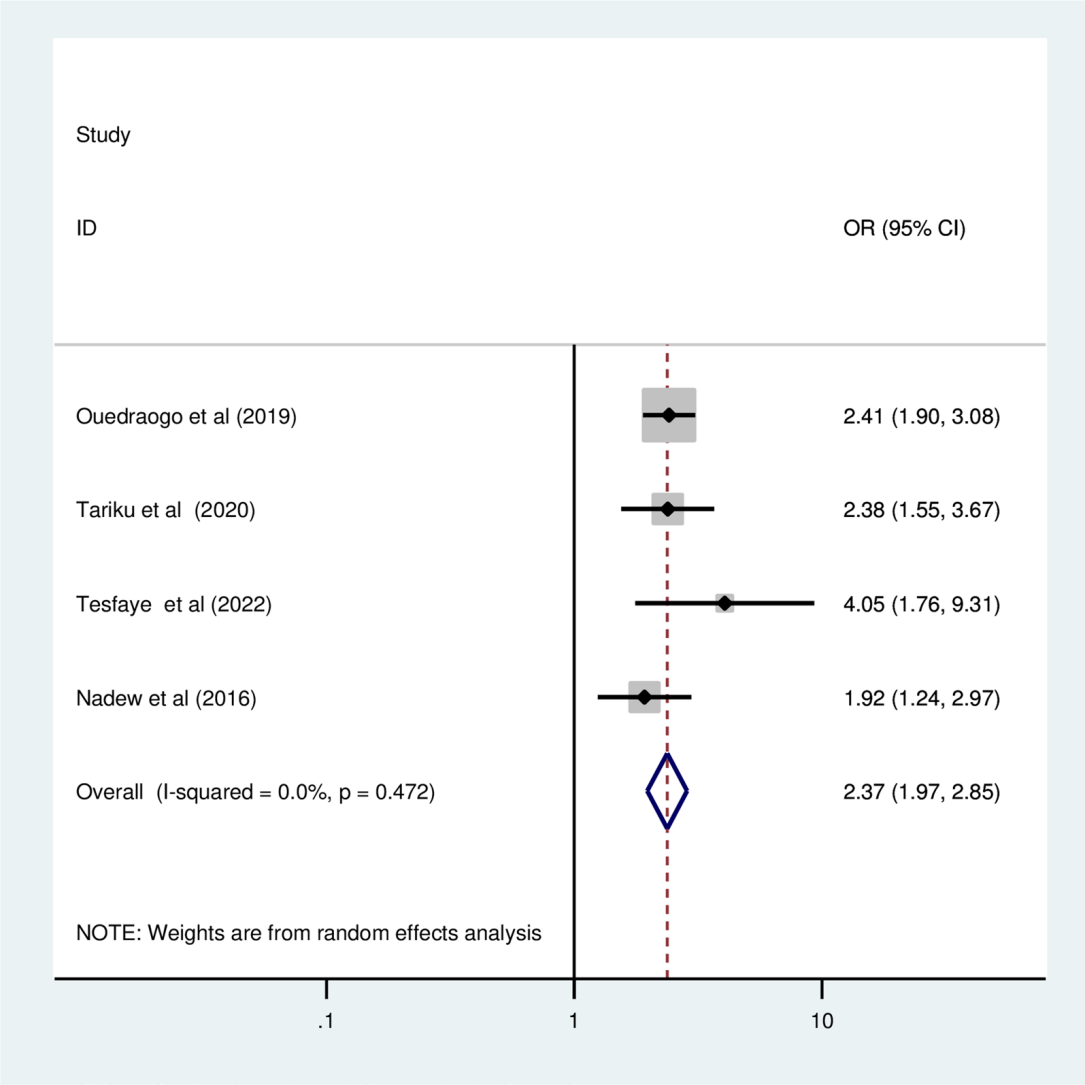
The pooled odd ratio of the association between pregnant women having an ANC for current pregnancy and ITN utilization among pregnant women in Ethiopia, 2022

Four primary studies with a total 4,965 participants of reported pregnant woman who had an ANC visits as a predictor of ITN utilization in pregnant women [10, 11, 13, 22]. The pooled odds ratio showed that a pregnant woman who had an ANC visits was 2.37 times more likely to utilize ITN than those who had not begun ANC (OR = 2.37, 95% CI 1.97, 2.65) (Fig. 9).

### Association of maternal level of knowledge on malaria prevention and ITN utilization

**Fig. 10:**
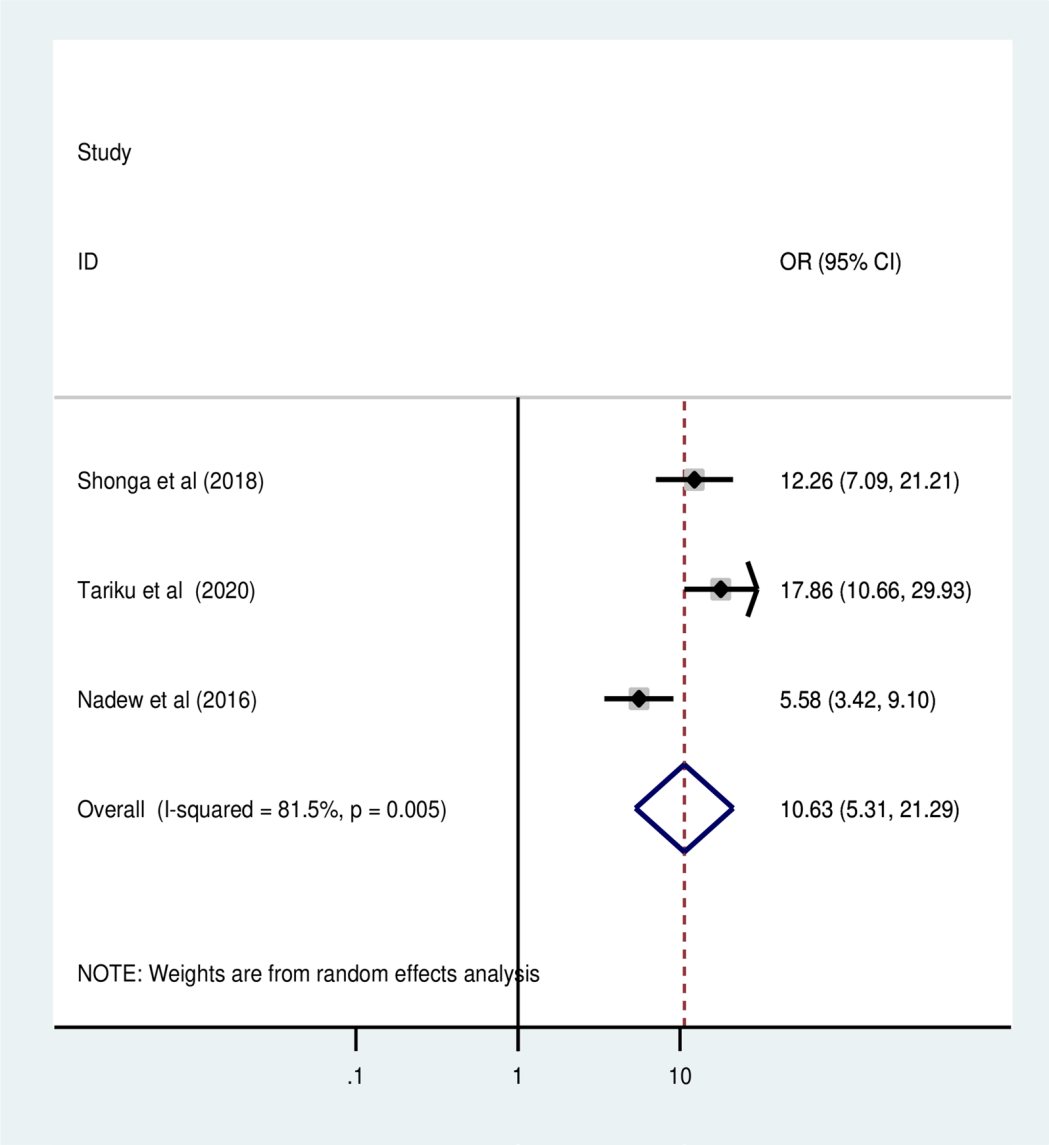
The pooled odd ratio of the association between having good level of knowledge on malaria prevention and ITN utilization among pregnant women in Ethiopia, 2022

Three primary studies with a total of 1,372 participants reported good level of knowledge on malaria prevention practice during pregnancy as a determinant of ITN utilization in pregnant women [10–12]. The odds ratio indicated that pregnant women with having good level of knowledge on malaria prevention practice during pregnancy had a 10.63 times higher chance of using ITN during pregnancy than those with poor knowledge (OR = 10.63, 95% CI 5.31, 21.29) (Fig. 10).

## Discussion

This systematic review and meta-analysis was aimed to estimate the pooled prevalence of ITN utilization and its associated factors among pregnant women in Ethiopia. Accordingly, the pooled prevalence of ITN utilization among pregnant women with at least one ITN in Ethiopia was 59.42% (95% CI 51.14, 67.7). The results of this meta-analysis was consistent with the findings from the 2015 national malaria indicators survey in Ethiopia (64.9%) [42], Kenya (55.8%) [24], a systematic review and meta-analysis of SSA (58.3%) [25] and Niger (65.1%) [26].

However, it was higher than a study in Cameron (12.4%) [27], Uganda (35%) [28], Ghana 2019 malaria indicator survey (49.2%) [29], and Nigeria (43.3%) [30], and lower than the studies reported from Kenya (70.5%) [31], Multi-level analysis from SSA (74.2%) [32], and the Democratic Republic of Congo (76.4%) [33]. This could be due to differences in sociodemographic characteristics, norms, beliefs and other cultural variations across the study settings. This discrepancy could also be explained by the differences in coverage and ITN ownership. Approximately 100% of respondents in the primary studies included in this review had at least one ITN. Only 59.7%, 52%, and 70.3% of the respondents owned ITNs in Nigeria and Ghana, respectively. Evidence indicates that higher rates of ITN ownership are directly correlated with better ITN utilization [32].

In the subgroup analysis, the utilization of ITN significantly varied across the subnational region of Ethiopia. The pooled proportion of pregnant women who slept under ITN was 74.56% in SNNPR, 60% in Tigray, 52.74% in Amhara and 47.62% in Oromia. Based on this subgroup analysis the highest ITN utilizers were from SNNPR (74.56%) and the lowest were from Oromia (47.62%). The differences between regions may be due to differences in temperature, environmental factors, malaria preventive strategies, and mosquito population density. Therefore, compared with areas with lower mosquito populations, ITN use is anticipated to be higher in areas with high mosquito populations [38, 39]. In addition, this could be due to the number of studies included in the analysis in which only two studies represented the Oromia regional state of Ethiopia.

According to a subgroup analysis by study setting there was substantial variation in the utilization of ITN by urban and rural pregnant women. The highest utilization of ITN among pregnant women was observed in studies conducted in urban setting (70.08%) compared to studies conducted in rural settings (56.72%). This may be because women from urban communities were closer to health professionals/health institutions, media, and education; there was a difference in ITN utilization by residence. Consequently, they may be aware of the risk of malaria and the benefits of sleeping under ITN during pregnancy [41].

A subgroup analysis by study period (malaria transmission season) was also performed to assess the seasonal variation in ITN utilization by pregnant women. The analysis indicated that ITN utilization among pregnant women in Ethiopia was higher among studies conducted during the high malaria transmission season as compared to studies conducted during the low malaria transmission season. This might be due to the fact that pregnant women during this high transmission season pregnant women may be more likely to use bed nets due greater awareness of the risk of malaria and the importance of prevention measures. In addition, during this season there may be more mosquitoes, which can increase the perceived importance of utilizing ITN.

This meta-analysis also aimed to identify factors associated with ITN utilization among pregnant women in Ethiopia. The current meta-analysis showed that higher ITN utilization was observed in pregnant women with a higher level of education. The odds of using ITN during pregnancy were 3.5 times higher among pregnant women with higher levels of education than among their counterparts. This result was consistent with findings from a meta-analysis conducted in Ghana [35], the Democratic Republic of Congo [34], and nations with a high malaria incidence [36]. The possible justification offered is that those with greater education are considered to have a high level of awareness of the value and significance of utilizing ITNs to prevent malaria [15].

This review also found that pregnant women with a good level of knowledge about malaria prevention had a 10.63 times higher chance of using ITN than those who had poor level of knowledge. Comparably, studies in the Democratic Republic of Congo [36], meta-analysis from malaria-endemic countries [34], Kenya [31] and meta-analysis from SSA countries [25] have revealed positive associations between the use of ITNs and adequate knowledge of various aspects of malaria among pregnant women in these countries. Therefore, to encourage the adherence of pregnant women to malaria control programs, behavioural change interventions should focus on sociocultural factors and universal education [40].

Based on this meta-analysis, a pregnant woman who had an ANC visits were 1.83 times more likely to use ITN than people who did not. This is in line with studies conducted in Uganda [28] and Demographic Republic of Congo [36]. This could be attributed to those who initially lacked an ITN receiving it from a health facility during an ANC visit after learning about its benefits. ITN users were also encouraged to use it because doing so would benefit both them and their unborn children. Warnings about the consequences of not utilizing an ITN for her and her unborn child may have also been given to achieve compliance. The possible explanation for this result could also be engagement in interpersonal communication workshops held for pregnant women during antenatal care.

## Strength and limitation of the study

This review has a number of strengths, including the following; to raise the standard of the review, thorough search techniques and the PRISMA checklist was used. While this review has some limitations, such as the fact that it only included research that were published in English. Additionally, because only studies from five regions of the country were included in this meta-analysis, the results may not be entirely representative. While malaria transmission intensity may explain differences in varying ITN utilization across the five regions of Ethiopia, subgroup analyses across different transmission strata were not conducted as not all studies reported reliable and current estimates of malaria transmission intensity. The exclusion of studies that were difficult to access, such as those published in languages other than English or those not available through online databases may limit the scope of this meta-analysis. The lack of an operational definition of good malaria prevention knowledge in the primary studies included may affect the results and conclusions. Lastly, some of the findings were also discussed with primary studies because there are only a few national and international systematic reviews and meta-analyses.

## Conclusion

This meta-analysis revealed that substantial proportion of pregnant women did not sleep under ITN during pregnancy. Only six tenth of pregnant women slept with an ITN the night before the survey which was much lower than the WHO recommendation (80%) [1]. Having higher education status, a pregnant woman who had an ANC visit and having a good level of knowledge on malaria prevention were found to be independent predictors of ITN utilization among pregnant women. Boosting women’s understanding of ITNs and malaria prevention will enhance their use, and the government and health sectors should encourage pregnant mothers to enroll in antenatal care. Furthermore, the implementation of sustainable behavioural change communication focusing on caring practices and regular utilization of ITNs after ITN distribution coordinated with prioritizing the most vulnerable groups is recommended.

## Supplementary Information

**Additional file 1: ** Searching strategy for systematic review and meta-analysis on Insecticide Treated Bed Net Utilization and Associated Factors among Pregnant Women in Ethiopia, 2022

**Additional file 2:** PRISMA 2020 Checklist for reporting the findings.

## Data Availability

All relevant data used for the systematic review and meta-analysis are within the manuscript and its supporting information.

## Abbreviations

ANC: Antenatal Care
CI: Confidence Interval
ITN: Insecticide Treated bed Net
IUGR: Intra Uterine Growth Restriction
JBI: Joanna Briggs Institute
OR: Odds Ratio
PRISMA: Preferred Reporting Items for Systematic Reviews and Meta-Analyses
SSA: Sub-Sahara Africa
SNNPR: Southern Nation Nationalities and People Region
-CS: Cross-sectional
