# Supplementary material for: Insecticide-treated bed net utilization and associated factors among pregnant women in Ethiopia: a systematic review and meta-analysis
Source: Malar J. 2023 Aug 2;22:223. doi: 10.1186/s12936-023-04655-7 (PMC10398969; doi:10.1186/s12936-023-04655-7)
Supplement: Supplementary file 1 — Additional file 1: Searching strategy for systematic review and meta-analysis on Insecticide Treated Bed Net Utilization and Associated Factors among Pregnant Women in Ethiopia, 2022 [file 12936_2023_4655_MOESM1_ESM.docx]

S2 File: Searching strategy for systematic review and meta-analysis on Insecticide Treated Bed Net Utilization and Associated Factors among Pregnant Women in Ethiopia, 2022

| **Database** | **Example of searching strategy** | **Number of studies** |
| --- | --- | --- |
| PubMed | ((((("Insecticide-treated bed net *") OR (Long-lasting insecticidal nets (LLINs)) OR (ITN)) AND ((((((("utilization"[All Fields]) OR ("use*"[All Fields])) OR ("practices"))) AND ((((((ethiopian) OR (ethiopia)) OR ("southern Ethiopia")) OR ("northern Ethiopia")) OR ("eastern Ethiopia")) OR ("western Ethiopia")) | 214 |
| Google Scholar | " insecticide-treated bed net" OR“ Long-lasting insecticidal nets (LLINs) AND "utilization " OR "use” OR “practices AND "associated factors" OR "determinates" AND “pregnant women” AND "Ethiopia" | 164 |
| AJOL | (“insecticide-treated bed net” OR “Long-lasting insecticidal nets (LLINs)” “bed net” and “utilization” AND Ethiopia | 11 |
| science direct | “insecticide-treated bed net utilization" AND "Ethiopia” | 41 |
| Cochrane Library | ("Insecticide-treated bed net [MeSH]" OR Long-lasting insecticidal nets (LLINs)" and utilization) AND Ethiopia | 21 |
| Unpublished (Addis Ababa university institutional repository ) | “insecticide-treated bed net OR Long-lasting insecticidal nets (LLINs) utilization" AND "Ethiopia” | 2 |
